# Patient characteristics associated with clinically coded long COVID: an OpenSAFELY study using electronic health records

**DOI:** 10.3399/BJGPO.2024.0140

**Published:** 2025-12-19

**Authors:** Yinghui Wei, Elsie MF Horne, Rochelle Knight, Genevieve Cezard, Alex J Walker, Louis Fisher, Rachel Denholm, Kurt Taylor, Venexia Walker, Stephanie Riley, Dylan M Williams, Robert Willans, Simon Davy, Sebastian Bacon, Ben Goldacre, Amir Mehrkar, Spiros Denaxas, Felix Greaves, Richard J Silverwood, Aziz Sheikh, Nish Chaturvedi, Angela M Wood, John Macleod, Claire Steves, Jonathan Sterne

**Affiliations:** 1 Centre for Mathematical Sciences, School of Engineering, Computing and Mathematics, University of Plymouth, Plymouth, UK; 2 Population Health Sciences, Bristol Medical School, University of Bristol, Bristol, UK; 3 Exeter Medical School, Faculty of Health and Life Sciences, University of Exeter, Exeter, UK; 4 Population Health Sciences, Bristol Medical School, University of Bristol, Bristol, UK; 5 NIHR Bristol Biomedical Research Centre, Bristol, UK; 6 MRC Integrative Epidemiology Unit, University of Bristol, Bristol, UK; 7 The National Institute for Health and Care Research Applied Research Collaboration West at University Hospitals Bristol and Weston NHS Foundation Trust, Bristol, UK; 8 Department of Public Health and Primary Care, University of Cambridge, Cambridge, UK; 9 The Bennett Institute for Applied Data Science, Nuffield Department of Primary Care Health Sciences, University of Oxford, Oxford, UK; 10 Health Data Research UK South-West, Bristol, UK; 11 Department of Surgery, University of Pennsylvania Perelman School of Medicine, Philadelphia, United States; 12 Centre for Mathematical Sciences, University of Plymouth, Plymouth, UK; 13 MRC Unit for Lifelong Health and Ageing, University College London (UCL), London, UK; 14 National Institute for Health and Care Excellence, London, UK; 15 Institute of Health Informatics, UCL, London, UK; 16 British Heart Foundation Data Science Centre, Health Data Research, London, UK; 17 NIHR UCL Hospitals Biomedical Research Centre (UCLH BRC), London, UK; 18 UCL BHF Research Accelerator, UCL, London, UK; 19 Department of Primary Care and Public Health, Imperial College London, London, UK; 20 Centre for Longitudinal Studies, UCL Social Research Institute, UCL, London, UK; 21 Usher Institute, The University of Edinburgh, Edinburgh, UK; 22 Department of Twin Research and Genetic Epidemiology, School of Life Course Sciences, King’s College London, London, UK

**Keywords:** COVID-19, SARS-CoV-2, Long COVID, post-acute COVID-19 syndrome

## Abstract

**Background:**

Clinically coded long COVID cases in electronic health records (EHRs) are incomplete, despite reports of rising cases of long COVID.

**Aim:**

To determine patient characteristics associated with clinically coded long COVID.

**Design & setting:**

With the approval of NHS England, we conducted a cohort study using EHRs within the OpenSAFELY-TPP platform in England, to study patient characteristics associated with clinically coded long COVID from 29 January 2020 to 31 March 2022.

**Method:**

We summarised the distribution of characteristics for people with clinically coded long COVID. We estimated age–sex adjusted hazard ratios (aHRs) and fully aHRs for coded long COVID. Patient characteristics included demographic factors, and health behavioural and clinical factors.

**Results:**

Among 17 986 419 adults, 36 886 (0.21%) were clinically coded with long COVID. Patient characteristics associated with coded long COVID included female sex, younger age (aged <60 years), obesity, living in less deprived areas, ever smoking, greater consultation frequency, and history of diagnosed asthma, mental health conditions, pre-pandemic post-viral fatigue, or psoriasis. These associations were attenuated following two doses of COVID-19 vaccines compared with before vaccination. Differences in the predictors of coded long COVID between the pre-vaccination and post-vaccination cohorts may reflect the different patient characteristics in these two cohorts rather than the vaccination status. Incidence of coded long COVID was higher in those with hospitalised COVID-19 than with those with non-hospitalised COVID-19.

**Conclusion:**

We identified variation in coded long COVID by patient characteristic. Results should be interpreted with caution as long COVID was likely under-recorded in EHRs.

## How this fits in

Electronic health records (EHRs) for long COVID are incomplete. It is important to understand the characteristics of people who have had their long COVID coded in EHRs. This study identified a set of patient characteristics associated with clinically coded long COVID. This includes the frequency of prior GP–patient interaction, sociodemographical variables, history of diagnosed diseases, and SARS-CoV-2 severity.

## Introduction

Long COVID,^1^ also known as post-acute sequelae of SARS-CoV-2 (PASC)^2^, or post-COVID-19 syndrome,^3^ is an overarching term for the persistent symptoms for weeks, months,^4^ or years, following the severe acute respiratory syndrome coronavirus 2 (SARS-CoV-2) infection. National Institute for Health and Care Excellence (NICE) guidance on supporting patients with long COVID includes assessing people with symptoms after acute SARS-CoV-2, investigations, and referrals.^5^

Understanding risk factors for long COVID is a public health priority. Counts and rates of people having a long COVID code in English primary care varied by demographic factors but also considerably by the practice clinical software system.^6^ UK longitudinal cohort studies reported that risk factors for having long COVID included increasing age, female sex, obesity, poor pre-pandemic general and mental health, and asthma.^7,8^

Previous electronic health records (EHRs) analyses were based on the study period from 1 February 2020 to 9 May 2021,^7^ during which 4189 long COVID cases were clinically coded. This represents considerable under-reporting, compared with the Office for National Statistics' (ONS') estimate of 1.0 million people with self-reported long COVID^9^ in the UK in May 2021. The usage of long COVID codes has improved with time.^10^ General practice services were encouraged to enhance their knowledge on assessing and referring patients with long COVID as set out in NHS actions on long COVID for 2021–2022.^11^

We conducted a cohort study within the OpenSAFELY-TPP database (https://www.opensafely.org/), which includes detailed linked data on around 24 million people registered with an English GP using TPP SystmOne EHR software (see ‘Data source’). We aimed to quantify associations of patient characteristics, including vaccination status, COVID-19 severity, and history of a range of disease diagnoses, with coded long COVID in English primary care.

## Method

### Data source

We used patient data from primary care records managed by the GP software provider, TPP SystmOne, covering around 40% of the population in England. These data include clinically coded long COVID, information on sociodemographics, pre-existing health conditions, and frequencies of GP–patient interactions, which may be consultations or any practice contacts. Data were linked to national SARS-CoV-2 testing records (Second Generation Surveillance System), vaccination data (National Immunisation Management Service), Index of Multiple Deprivation (IMD), and the ONS death registry. Admitted Patient Care Spells (APCS) is part of Hospital Episode Statistics (HES) and is provided to OpenSAFELY via NHS Digital’s Secondary Uses Service (SUS). OpenSAFELY includes pseudonymised data such as coded diagnoses, medications, and physiological parameters, but does not include free-text data.

### Study population and cohort definitions

Our study population consisted of adults aged between 18 years and 105 years, with known sex and region, who were registered as active patients in a TPP GP on 29 January 2020 (the date when the first two SARS-CoV-2 cases were reported in the UK) and had at least 1 year of prior follow-up in a general practice, to ensure that baseline characteristics could be adequately captured.

We constructed four cohorts (Supplement, Figure S1, Table S1): (1) a primary general population cohort, with follow-up start date on 29 January 2020 and end date the earliest of first record of any long COVID code, death date, or 31 March 2022 (the day before free SARS-CoV-2 testing in England ended;^12^ (2) a post-COVID diagnosis cohort, defined regardless of vaccination status, with follow-up start date the first recorded COVID-19 diagnosis and end date the earliest of first record of any long COVID code, death date, or 31 March 2022; (3) a pre-vaccination cohort with follow-up start date on 29 January 2020 and end date the earliest of first record of any long COVID code, date of receipt of first COVID-19 vaccine dose, death date, or 31 March 2022; (4) a post-vaccination cohort, with follow-up start date 14 days after receipt of second COVID-19 vaccine dose and end date the earliest of first record of any long COVID code, death date, or 31 March 2022. In each cohort, people with a history of SARS-CoV-2 infection, and/or long COVID code before their follow-up start date, were excluded.

### Outcomes

The outcome was clinically coded long COVID, constructed from the date of the first record of any of the 15 UK SNOMED-CT codes for long COVID^6^ in English primary care records, consisting of two diagnostic codes, three referral codes, and 10 assessment codes (Supplement, Table S2). Time to the outcome event was defined as days from participant specific follow-up start date (Supplement, Table S1).

### COVID-19 diagnosis

Date of COVID-19 diagnosis was defined as the earliest of: record of a positive SARS-CoV-2 polymerase chain reaction or antigen test; confirmed COVID-19 diagnosis in primary care or secondary care hospital admission records; or death certificate with SARS-CoV-2 infection listed as primary or underlying cause.

### Patient characteristics

Patient characteristics included demographic variables, and health behavioural and clinical factors that may be associated with coded long COVID,^6,7^ and the frequency of GP–patient interactions, which could be an indicator of patient access to care and ability to interact with general practice. There is only one entry for sex in the EHR for each patient. All other coded values were the latest record on or before the cohort and participant specific follow-up start date. A full description of patient characteristics is in the Supplement, Table S2.

Demographic variables included age, sex, obesity, ethnicity, region, and deprivation. Where categorised, age groups were: 18–39 years, 40–59 years, 60–79 years, 80–105 years. Obesity was grouped based on body mass index (BMI kg/m^2^) using categories derived from the World Health Organization (WHO):^13^ no evidence of obesity BMI<30 kg/m^2^; obese class I, BMI 30–34.9 kg/m^2^; obese class II, BMI 35–39.9 kg/m^2^; and obese class III, BMI≥40 kg/m^2^. Ethnic groups were White, Mixed, Asian or Asian British, Black or Black British, and Chinese or other ethnic groups. All nine regions in England were included (East, London, East Midlands, North East, North West, West Midlands, Yorkshire and the Humber, South East, and South West).^14^ IMD was determined based on residential area categorised into five quintiles based on relative disadvantage, with quintile 1 (Q1) being the most deprived, and quintile 5 (Q5) being the least deprived.

Health behavioural and clinical factors included smoking status, frequency of GP–patient interaction and history of disease diagnoses. Smoking status was grouped into current-, ever-, and never-smokers. Frequency of GP–patient interaction was defined during the 12 months before participants’ follow-up start date, and categorised as: without any interaction; 1–3; 4–8; 9–12, and ≥13 interactions. History of the disease diagnoses, chosen based on previous literature on risk factors for long COVID^7^ and defined on or before the cohort and participant specific follow-up start date, was coded as separate indicator variables: asthma, cancer, chronic cardiac disease, chronic kidney disease, chronic liver disease, chronic obstructive pulmonary disease (COPD), chronic respiratory disease, dementia, diabetes, dysplenia (dysfunctional spleen), haematological cancer, heart failure, hypertension, mental health condition, organ transplant, other immunosuppressive condition, other neurological condition, post-viral fatigue, psoriasis, rheumatoid arthritis, systemic lupus erythematosus (SLE), and stroke. History of diagnosed post-viral fatigue was defined before 29 January 2020 owing to the potential use of the corresponding codes as a proxy for long COVID before the introduction of long COVID clinical codes in December 2020.

Hospitalisation for COVID-19 was defined as a hospital admission record with confirmed COVID-19 diagnosis in primary position within 28 days of the first COVID-19 diagnosis and COVID-19 without hospitalisation as a COVID-19 diagnosis that was not followed by hospitalisation within 28 days.^15^

### Statistical analyses

Rates of coded long COVID were quantified as the number of first long COVID events per 1000 person-years. The cumulative probability of coded long COVID was estimated, using the Kaplan–Meier approach, by age group and sex. In each cohort, hazard ratios with 95% confidence intervals (CIs) for each patient characteristic were estimated from age-and-sex adjusted Cox proportional hazards (PH) models, and then all patient characteristics were included in a multivariable Cox PH model. Age was modelled using a restricted cubic spline, and estimated log hazard ratios against continuous age were plotted. In the post-COVID diagnosis cohort, we included COVID-19 severity (hospitalised versus non-hospitalised COVID-19) as an additional factor. Hazard ratios by age group (40–59 years, 60–79 years, and 80–105 years compared with 18–39 years [reference]), were estimated from models including age as a categorical variable, instead of a cubic spline.

For computational efficiency, we used the full population with coded long COVID and a randomly sampled population without coded long COVID with a ratio of 1:20. We used inverse probability weighting and robust standard errors to account for the sampling approach. The discriminative ability of the fitted model was quantified using C-statistics.^16^

We included a missing category for ethnicity, smoking status, and IMD. All other covariates were defined using the presence versus absence of specific codes, and thus have no identifiable missing values.

Data management and analysis were conducted using Python (version 3.8) and R (version 4.2.1) according to a prespecified protocol. Our protocol, analysis code, and code lists are available.^17^

## Results

### Study population

In total, 17 986 419 adults were included in the primary and pre-vaccination cohorts, 13 401 208 in the post-vaccination cohort and 3 507 738 in the post-COVID diagnosis cohort (Table 1). In the primary cohort, there were missing data for ethnicity (4 809 699, 26.74%), smoking status (744 851, 4.14%), and IMD (298 586, 1.66%). There were 1 855 613 (10.32%) people with ethnicity recorded as from minority groups, including Asian or Asian British, Black or Black British, Chinese or other ethnic groups, or Mixed. People in the post-vaccination and post-COVID diagnosis cohorts were more likely to have had at least one GP interaction 12 months before follow-up than those in the primary cohort. In each cohort, the most prevalent previous diagnoses were of asthma, chronic cardiac disease, diabetes, hypertension, and mental health conditions. People in the post-vaccination cohort were older, less likely to be recorded as from a minority ethnic group, and more likely to have a history of prior disease diagnoses than those in the pre-vaccination cohort. People in the post-COVID diagnosis cohort were younger, more likely to be male, and more likely to be recorded as from a minority ethnic group than those in the primary cohort. This motivates future research on increasing vaccine uptake in minority ethnic groups.

**Table 1.** Patient characteristics. Summary statistics are number (percentage) except where indicated

		Cohort		
**Characteristic**		**Primary** or **pre-vaccination**	**Post-vaccination**	**Post-COVID diagnosis**
All		17 986 419	13 401 208	3 507 738
Mean (SD) age, years		49.72 (18.69)	53.45 (18.40)	44.55 (17.26)
Age, years	18–39	6 163 161 (34.27)	3 433 136 (25.62)	1 508 578 (43.01)
	40–59	6 143 985 (34.16)	4 732 533 (35.31)	1 340 894 (38.23)
	60–79	4 513 280 (25.09)	4 162 621 (31.06)	515 422 (14.69)
	80–105	1 165 993 (6.48)	1 072 918 (8.01)	142 844 (4.07)
Sex	Female	8 971 008 (49.88)	6 451 356 (48.14)	1 601 255 (45.65)
	Male	9 015 411 (50.12)	6 949 852 (51.86)	1 906 483 (54.35)
BMI	Not obese	13 826 227 (76.87)	9 964 252 (74.35)	2 617 431 (74.62)
	Obese i (30–34.9)	2 602 204 (14.47)	2 128 044 (15.88)	528 931 (15.08)
	Obese ii (35–39.9)	988 672 (5.50)	819 465 (6.11)	221 223 (6.31)
	Obese iii (≥40)	569 316 (3.17)	489 447 (3.65)	140 153 (4.00)
Ethnicity	White	11 321 107 (62.94)	9 100 727 (67.91)	2 290 746 (65.31)
	Asian or Asian British	1 056 550 (5.87)	678 284 (5.06)	228 256 (6.51)
	Black or Black British	345 940 (1.92)	183 283 (1.37)	67 597 (1.93)
	Chinese or other ethnic group	277 598 (1.54)	135 311 (1.01)	33 780 (0.96)
	Mixed	175 525 (0.98)	98 685 (0.74)	37 618 (1.07)
	Missing	4 809 699 (26.74)	3 204 918 (23.92)	849 741 (24.22)
Region	East	4 152 253 (23.09)	3 137 907 (23.42)	779 917 (22.23)
	East Midlands	3 116 231 (17.33)	2 358 504 (17.60)	641 785 (18.30)
	London	1 190 596 (6.62)	643 154 (4.80)	195 859 (5.58)
	North East	864 297 (4.81)	647 350 (4.83)	202 114 (5.76)
	North West	1 608 661 (8.94)	1 235 875 (9.22)	374 128 (10.67)
	South East	1 214 019 (6.75)	920 943 (6.87)	201 174 (5.74)
	South West	2 502 709 (13.91)	2 047 734 (15.28)	398 249 (11.35)
	West Midlands	728 973 (4.05)	475 426 (3.55)	153 392 (4.37)
	Yorkshire and the Humber	2 608 680 (14.50)	1 934 315 (14.43)	561 120 (16.00)
Index of Multiple Deprivation	1 (most deprived)	3 419 935 (19.01)	2 175 145 (16.23)	721 264 (20.56)
	2	3 504 610 (19.48)	2 450 814 (18.29)	695 034 (19.81)
	3	3 761 299 (20.91)	2 843 309 (21.22)	699 051 (19.93)
	4	3 666 169 (20.38)	2 900 059 (21.64)	683 882 (19.50)
	5 (least deprived)	3 335 820 (18.55)	2 755 089 (20.56)	624 221 (17.80)
	0 (missing)	298 586 (1.66)	276 792 (2.07)	84 286 (2.40)
Smoking status	Never smoker	8 257 661 (45.91)	6 221 555 (46.43)	1 681 507 (47.94)
	Current smoker	3 043 874 (16.92)	1 945 883 (14.52)	508 586 (14.5)
	Ever smoker	5 940 033 (33.03)	4 753 679 (35.47)	1 125 675 (32.09)
	Missing	744 851 (4.14)	480 091 (3.58)	191 970 (5.47)
GP–patient interaction	0	4 887 100 (27.17)	2 874 697 (21.45)	787 607 (22.45)
	1–3	4 481 433 (24.92)	3 535 283 (26.38)	970 395 (27.66)
	4–8	2 364 870 (13.15)	1 887 389 (14.08)	467 316 (13.32)
	9–12	4 517 010 (25.11)	3 682 594 (27.48)	935 897 (26.68)
	≥13	1 736 006 (9.65)	1 421 245 (10.61)	346 523 (9.88)
History of disease diagnosis	Asthma	3 039 981 (16.90)	2 366 051 (17.66)	693 139 (19.76)
	Cancer	898 197 (4.99)	830 573 (6.20)	131 263 (3.74)
	Chronic cardiac disease	1 216 263 (6.76)	1 083 971 (8.09)	188 628 (5.38)
	Chronic kidney disease	25 440 (0.14)	21 285 (0.16)	8085 (0.23)
	Chronic liver disease	104 376 (0.58)	90 055 (0.67)	18 422 (0.53)
	Chronic obstructive pulmonary disease	597 757 (3.32)	526 800 (3.93)	94 218 (2.69)
	Chronic respiratory disease	734 352 (4.08)	643 188 (4.80)	111 337 (3.17)
	Dementia	42 978 (0.24)	31 376 (0.23)	12 182 (0.35)
	Diabetes	1 827 304 (10.16)	1 678 045 (12.52)	322 903 (9.21)
	Dysplenia	25 815 (0.14)	22 137 (0.17)	4316 (0.12)
	Hematological cancer	104 655 (0.58)	95 011 (0.71)	19 085 (0.54)
	Heart failure	315 575 (1.75)	292 006 (2.18)	56 106 (1.60)
	Hypertension	3 845 579 (21.38)	3 409 919 (25.44)	563 055 (16.05)
	Mental health	3 677 686 (20.45)	2 946 356 (21.99)	784 619 (22.37)
	Organ transplant	20 848 (0.12)	17 921 (0.13)	5732 (0.16)
	Other immunosuppressive condition	90 212 (0.50)	78 650 (0.59)	20 283 (0.58)
	Other neurological disease	178 921 (0.99)	153 470 (1.15)	32 107 (0.92)
	Pre-pandemic post-viral fatigue	33 616 (0.19)	28 482 (0.21)	5912 (0.17)
	Psoriasis	697 571 (3.88)	575 811 (4.30)	143 676 (4.10)
	Rheumatoid arthritis	183 330 (1.02)	165 080 (1.23)	32 030 (0.91)
	Systematic lupus erythematosus	30 177 (0.17)	25 819 (0.19)	5495 (0.16)
	Stroke	381 038 (2.12)	339 592 (2.53)	62 944 (1.79)

BMI = body mass index. SD = standard deviation

The numbers of people with coded long COVID were 36 886 (0.2%), 7155 (0.04%), 17 376 (0.1%), and 29 268 (0.8%) in the primary, pre-vaccination, post-vaccination, and post-COVID diagnosis cohorts, respectively (Table 2). The corresponding incidence rates of coded long COVID were 1.0, 0.3, 1.6, and 12.8 per 1000 person-years, respectively. In the primary cohort, the rate was highest in people aged 40–59 years (1.4), females (1.2), and people with BMI >40 kg/m^2^ (1.8). In the post-COVID diagnosis cohort, the incidence rate was highest in people aged 40–59 years (17.0), females (14.8), and people with BMI >40 kg/m^2^ (20.2), of White ethnicity (14.0), and living in less deprived areas (IMD Q4: 14.7).

**Table 2.** Event count per 1000 person-years (pyrs) and incidence rate (IR) per 1000 person-years for long COVID

		Cohort							
		**Primary**		**Pre-vaccination**		**Post-vaccination**		**Post-COVID diagnosis**	
**Characteristic**		**Count/1000 pyrs**	**IR**	**Count/1000 pyrs**	**IR**	**Count**	**IR**	**Count/1000 pyrs**	**IR**
All		36 886/38 520.8	1.0	7155/23 609.2	0.3	17 376/11 142.9	1.6	29 268/2293.2	12.8
Age, years	18–39	12 031/13 368.9	0.9	3405/9659.6.6	0.4	4446/2366.3.3	1.9	9181/1000.5	9.2
	40–59	18 661/13 287.1	1.4	3092/7882.5.5	0.4	9218/3917.5.5	2.4	14 886/876.1	17.0
	60–79	5713/9611.4.4	0.6	616/4946.9.9	0.1	3371/3 805.5.5	0.9	4794/334.4	14.3
	80–105	481/2253.5.5	0.2	42/1120.2.2	0.0	341/1053.6.6	0.3	407/82.1	5.0
Sex	Male	13 569/19 209.6	0.7	2803/12 151.4	0.2	6440/5263.6.6	1.2	10 836/1047.4	10.3
	Female	23 317/19 311.3	1.2	4352/11 457.8	0.4	10 936/5879.4.4	1.9	18 432/1245.8	14.8
BMI	Not obese	24 736/29 616.8	0.8	5203/18 604.8	0.3	11 685/8179.8.8	1.4	19 045/1694.2	11.2
	Obese i (30–34.9 kg/m²)	6881/5570.8.8	1.2	1138/3146.3.3	0.4	3235/1832.8.8	1.8	5679/354.3	16.0
	Obese ii (35–39.9 kg/m²)	3083/2116.4.4	1.5	497/1187.6.6	0.4	1437/702.7	2.0	2627/149.9	17.5
	Obese iii (≥40 kg/m²)	2186/1216.8.8	1.8	317/670.6	0.5	1019/427.7	2.4	1917/94.7	20.2
Ethnicity	White	24,553/24,226.3	1.0	4,363/14,430.7	0.3	12,385/7,682.1.1	1.6	20,045/1,436.2	14.0
	Asian or Asian British	2172/2279.4.4	1.0	598/1519.2.2	0.4	612/525.8	1.2	1795/190.3	9.4
	Black or Black British	544/746.1	0.7	244/545.4	0.4	138/140.7	1.0	428/48.2	8.9
	Chinese or other ethnic group	289/600.6	0.5	87/461.8	0.2	107/104.1	1.0	222/23.4	9.5
	Mixed	345/379.4	0.9	133/274.7	0.5	105/75.6	1.4	276/25.6	10.8
	Missing	8983/10 289.0	0.9	1730/6377.4.4	0.3	4029/2614.6.6	1.5	6502/569.4	11.4
Region	East	6476/8897.2.2	0.7	1225/5428.5.5	0.2	3061/2600.3.3	1.2	4744/499.2	9.5
	East Midlands	5100/6671.6.6	0.8	1048/4035.3.3	0.3	1928/1959.6.6	1.0	4066/425.2	9.6
	London	1500/2566.1.1	0.6	476/1854.5.5	0.3	486/512.7	0.9	974/131.9	7.4
	North East	3484/1848.2.2	1.9	495/1111.4.4	0.4	1858/542.7	3.4	3067/137.0	22.4
	North West	4329/3438.5.5	1.3	704/2037.2.2	0.3	2269/1035.1.1	2.2	3451/251.9	13.7
	South East	2824/2598.3.3	1.1	763/1581.8.8	0.5	1051/772.5	1.4	2308/123.1	18.7
	South West	5329/5355.4.4	1.0	879/3138.3.3	0.3	3150/1718.2.2	1.8	4244/227.8	18.6
	West Midlands	1223/1561.0.0	0.8	329/1011.7.7	0.3	454/392.3	1.2	909/110.9	8.2
	Yorkshire and The Humber	6621/5584.6.6	1.2	1236/3410.5.5	0.4	3119/1609.5.5	1.9	5505/386.3	14.3
Index of Multiple Deprivation	1 (most deprived)	6529/7318.8.8	0.9	1656/4828.4.4	0.3	2392/1753.7.7	1.4	5254/509.3	10.3
	2	6964/7503.8.8	0.9	1450/4745.7.7	0.3	3011/2013.6.6	1.5	5522/466.5	11.8
	3	7430/8054.1.1	0.9	1449/4890.9.9	0.3	3429/2375.3.3	1.4	5877/448.6	13.1
	4	8185/7853.4.4	1.0	1371/4 641.4.4	0.3	4255/2442.7.7	1.7	6357/431.9	14.7
	5 (least deprived)	7135/7150.1.1	1.0	1108/4112.1.1	0.3	3907/2333.9.9	1.7	5629/385.5	14.6
	Missing	643/640.6	1.0	121/390.7	0.3	382/223.7	1.7	629/51.4	12.2

BMI = body mass index. IR = incidence rate. Pyrs = person-years

In the primary cohort, the overall cumulative probability of coded long COVID was less than 0.1% in people aged ≥80 years, rising to around 0.4% and 0.2%, respectively, in women and men aged 40–59 years (Supplement, Figure S2). In the post-COVID diagnosis cohort, the overall cumulative probability of coded long COVID was <0.5% in people aged ≥80 years, rising to around 1.3% and 0.9%, respectively, in women and men aged 40–59 years (Supplement, Figure S3). The low cumulative probability of coded long COVID for people aged ≥80 years may have been owing to higher risk of mortality, which can censor diagnosis of long COVID.

### Demographic factors: primary and post-COVID diagnosis cohorts

**Figure 1.**
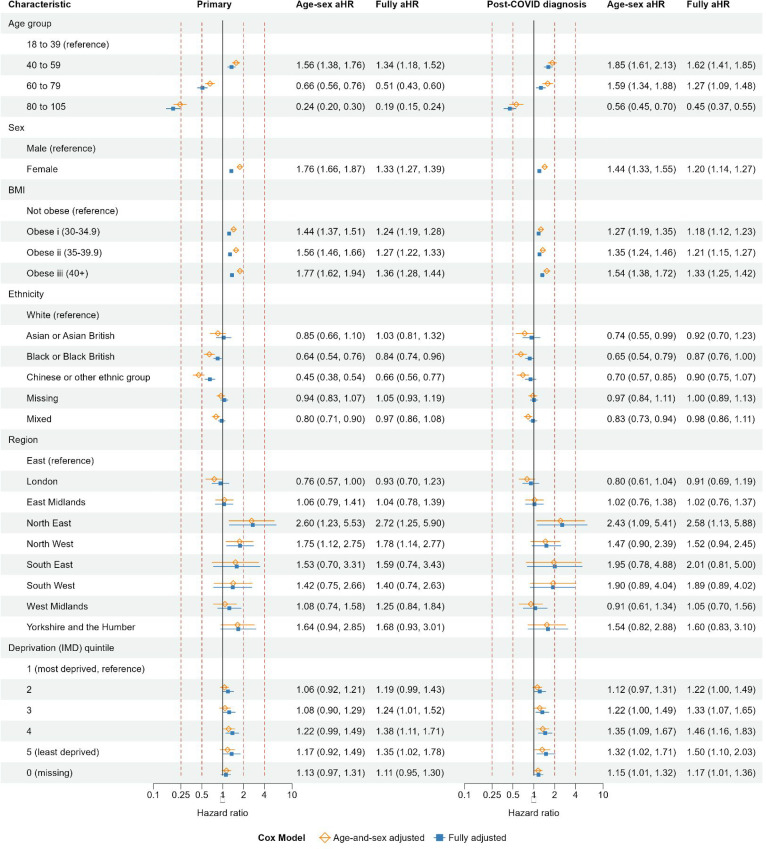
Primary and post-COVID diagnosis cohorts: age-and-sex adjusted and fully adjusted hazard ratios of coded long COVID for demographic variables. BMI = body mass index. IMD = Index of Multiple Deprivation

Fully adjusted hazard ratios (aHRs) for sex, obesity, and ethnicity were generally attenuated towards 1, compared with age–sex aHR (Figure 1). The incidence of coded long COVID declined markedly with age in the primary cohort (aHRs 0.51 [95% confidence interval {CI} = 0.43 to 0.60]) and 0.19 (95% CI = 0.15 to 0.24) for age groups 60–79 and 80–105 years, respectively, compared with age group 18–39 years). This decline was less marked in the post-COVID diagnosis cohort. The aHRs comparing age groups were consistent with those when age was modelled by restricted cubic spline (supplement Figure S4). The incidence of coded long COVID was higher in females than males in (aHRs 1.33 [95% CI = 1.27 to 1.39]) and 1.20 [95% CI = 1.14 to 1.27] in the primary and post-COVID diagnosis cohorts, respectively). In the primary cohort, the incidence of coded long COVID was lower in people from Black or Black British ethnicity (aHR 0.84 [95% CI = 0.74 to 0.96]) and Chinese or other ethnic groups (aHR 0.66 [95% CI = 0.56 to 0.77]), compared with those of White ethnicity. These differences were attenuated towards 1 in the post-COVID diagnosis cohort. In each cohort, the incidence of coded long COVID was higher in North East, and increased with increasing obesity and decreasing deprivation.

### Demographic factors: pre-vaccination and post-vaccination cohorts

**Figure 2.**
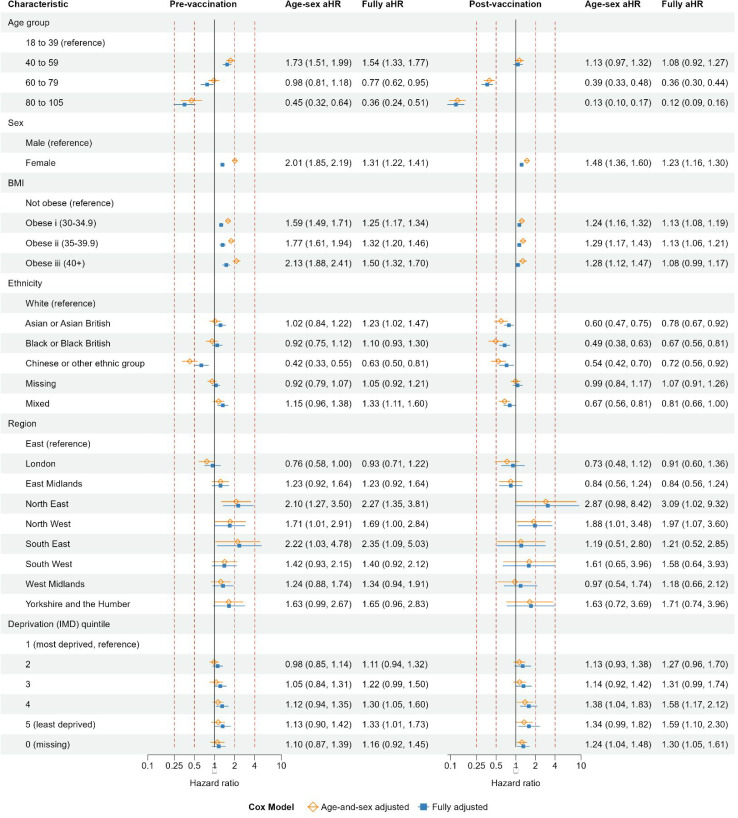
Pre-vaccination and post-vaccination cohorts: age-and-sex adjusted and fully adjusted hazard ratios of coded long COVID for demographic variables. BMI = body mass index. IMD = Index of Multiple Deprivation

Fully aHRs for sex and BMI were generally attenuated towards 1, compared with age–sex aHRs (Figure 2) in both pre-vaccination and post-vaccination cohorts. The incidence of coded long COVID declined in older adults in the post-vaccination cohort (aHRs 0.36 [95% CI = 0.30 to 0.44] and 0.12 [95% CI = 0.09 to 0.16] for age groups 60–79 years and 80–105 years, respectively, compared with younger adults aged 18–39 years). This decline was less marked in the pre-vaccination cohort. The incidence of coded long COVID was higher in females than males (aHRs 1.31 [95% CI = 1.22 to 1.41] and 1.23 [95% CI = 1.16 to 1.30] in the pre-vaccination and post-vaccination cohorts, respectively). In the pre-vaccination cohort, the incidence of coded long COVID was increased with increasing obesity. This pattern was less clear in the post-vaccination cohort. In both cohorts, the incidence of coded long COVID was lower in people of Chinese or other ethnic groups (aHRs 0.63 [95% CI = 0.50 to 0.81] and 0.72 [95% CI = 0.56 to 0.92] in the pre-vaccination and post-vaccination cohorts, respectively), compared with those of White ethnicity. The incidence of coded long COVID was lower in people of Black or Black British ethnicity compared with White ethnicity in the post-vaccination cohort (aHR 0.67 [95% CI = 0.56 to 0.81]), but not in the pre-vaccination cohort (aHR 1.10 [95% CI = 0.93 to 1.30]). In the pre-vaccination cohort, the incidence of coded long COVID was slightly higher in North East. In the post-vaccination cohort, it was slightly higher in North West. In each cohort, the incidence of long COVID increased with decreasing deprivation.

### Health behavioural and clinical factors: primary and post-COVID diagnosis cohorts

**Figure 3.**
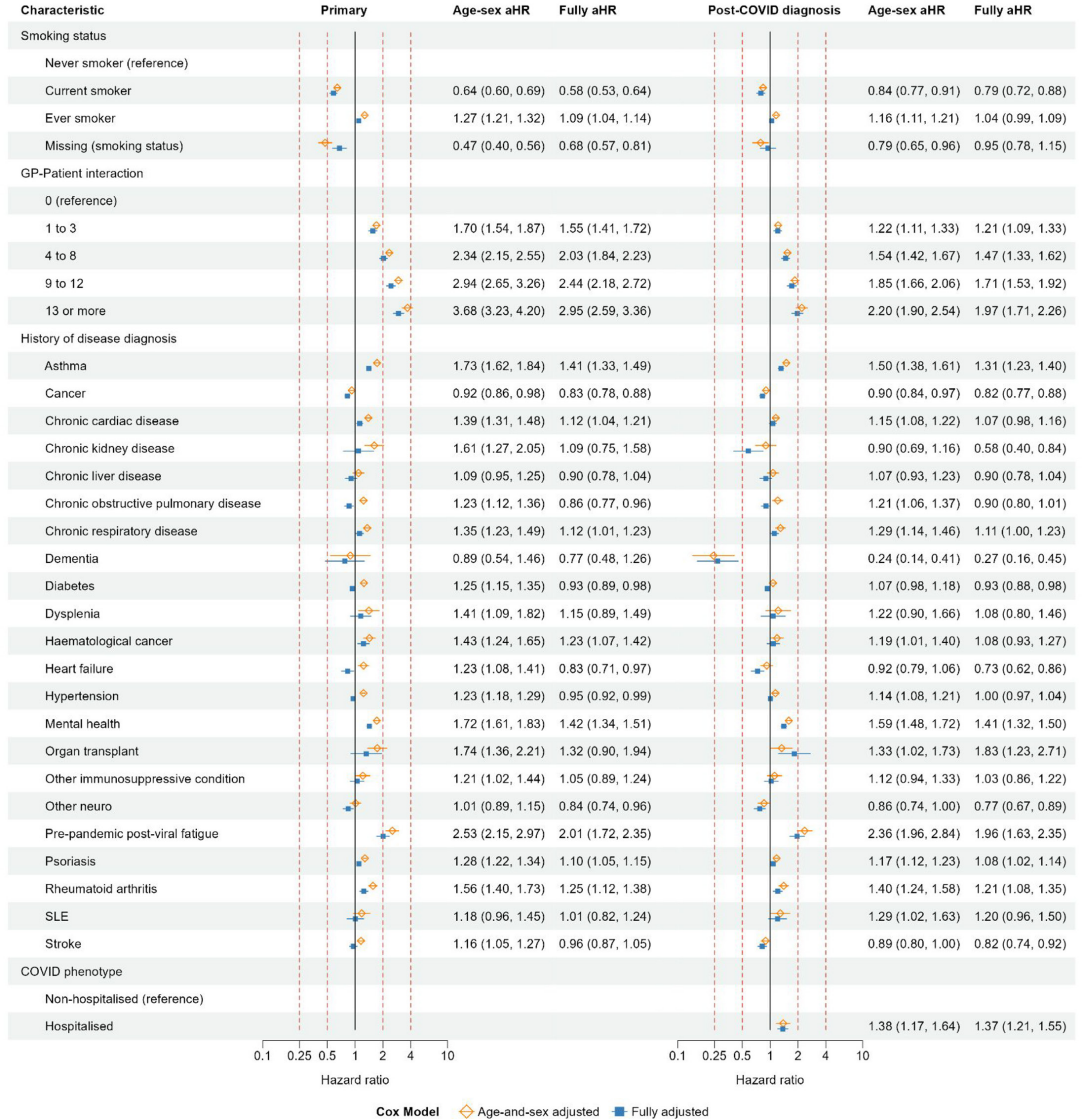
Primary and post-COVID diagnosis cohorts: age-and-sex adjusted and fully adjusted hazard ratios of coded long COVID for health behavioural and clinical variables. SLE = systemic lupus erythematosus

In the primary cohort, the incidence of coded long COVID was lower in current smokers and people with a missing smoking status, compared with people who never smoked (Figure 3). These differences were attenuated towards 1 in the post-COVID diagnosis cohort. In each cohort, the incidence of coded long COVID increased with increasing frequency of GP–patient interactions, during 12 months before the follow-up start date. The aHRs for GP–patient interaction were generally attenuated, compared with age–sex aHRs.

In the primary cohort, the incidence of coded long COVID was higher in people with than without a history of diagnosed asthma, chronic cardiac disease, chronic respiratory disease, haematological cancer, mental health conditions, pre-pandemic post-viral fatigue, psoriasis, or rheumatoid arthritis. These differences were generally attenuated in the post-COVID diagnosis cohort. In both cohorts, aHRs for these diseases were attenuated towards 1, compared with age–sex aHRs. The largest aHRs were for pre-pandemic post-viral fatigue (pre-vaccination cohort: 2.01, 95% CI = 1.72 to 2.35; post-vaccination cohort: 1.96, 95% CI = 1.63 to 2.35). In the primary cohort, the incidence of coded long COVID was lower in people with than without a history of diagnosed cancer, COPD, diabetes, heart failure, hypertension, or other neurological disorders. In the post-COVID diagnosis cohort, incidence of coded long COVID was similar in people with and without a history of diagnosed hypertension (aHR 1.00 [95% CI = 0.97 to 1.04]). In the post-COVID diagnosis cohort, people with hospitalised COVID-19 had higher incidence of coded long COVID (aHR 1.37 [95% CI = 1.21 to 1.55]) than those with non-hospitalised COVID-19.

### Health behavioural and clinical factors: pre-vaccination and post-vaccination cohorts

**Figure 4.**
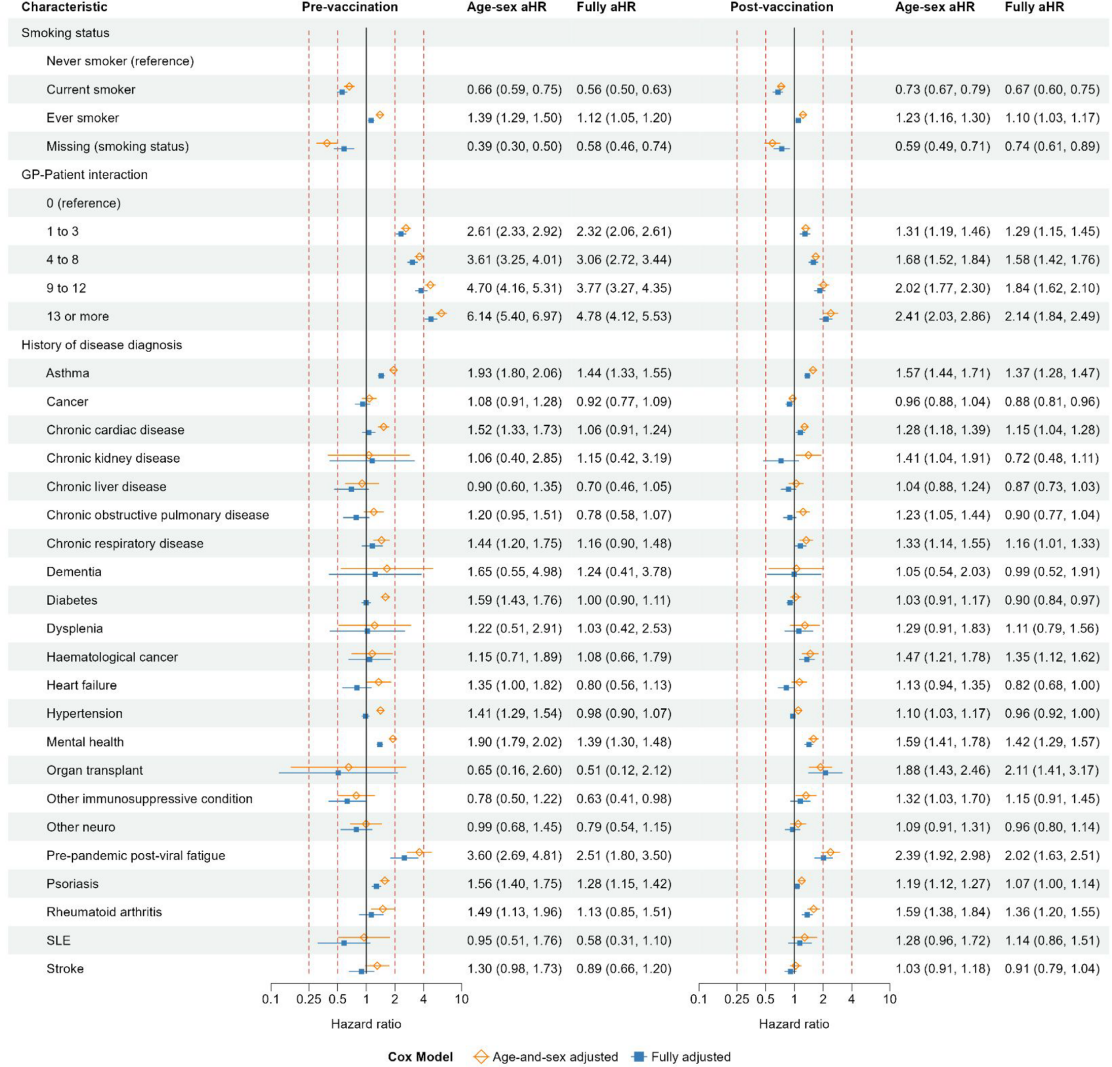
Pre-vaccination and post-vaccination cohorts: age-and-sex adjusted and fully adjusted hazard ratios of coded long COVID for health behavioural and clinical variables. SLE = systemic lupus erythematosus

In the pre-vaccination cohort, the incidence of coded long COVID was lowest in current smokers and people with a missing smoking status, and highest in ever smokers, compared with people who never smoked (Figure 4). The aHRs for smoking status were attenuated towards 1 in the post-vaccination cohort, compared with the pre-vaccination cohort. The incidence of coded long COVID increased with increasing frequency of GP–patient interaction, although aHRs were attenuated towards 1 in the post-vaccination cohort, compared with the pre-vaccination cohort. The aHRs for GP–patient interaction were generally attenuated, compared with age–sex adjusted hazard ratios.

In the pre-vaccination cohort, the incidence of coded long COVID was higher in people with than without a history of diagnosed asthma, mental health conditions, pre-pandemic post-viral fatigue, and psoriasis. These differences were attenuated in the post-vaccination cohort, compared with the pre-vaccination cohort. The aHRs for these diseases were attenuated, compared with age–sex aHRs. In the post-vaccination cohort, but not the pre-vaccination cohort, the incidence of coded long COVID was higher in people with than without a history of organ transplant. The incidence of coded long COVID was higher in people with than without a history of diagnosed pre-pandemic post-viral fatigue, in both the pre-vaccination and post-vaccination cohorts.

## Discussion

### Summary

Despite an estimated 2.8% of the UK population having self-reported symptoms of long COVID^18^ as of 3 April 2022, only 36 886 (0.2%) of the eligible general adult population in this study of 17 986 419 adults had a diagnosis of long COVID recorded in their primary care record.

Patient characteristics associated with higher incidence of coded long COVID included female sex, younger age (aged <60 years), greater BMI, ever having smoked, and a history of diagnosed asthma, mental health conditions, and psoriasis. The incidence of coded long COVID was higher with increasing GP–patient interaction. Coded long COVID was more than twice as likely in people with than without a diagnosis of post-viral fatigue before the pandemic. The incidence of coded long COVID was higher after hospitalised than non-hospitalised COVID-19.

Differences between factors associated with coded long COVID in the four cohorts studied may reflect differences between risk factors for infection with SARS-CoV-2, developing severe COVID-19, and developing long COVID having been infected with SARS-CoV-2. They may also reflect the influence of vaccination on developing long COVID, and changes in primary care coding practice and healthcare-seeking behaviours during the pandemic. There were only minor differences between the cohorts in associations of demographic factors with coded long COVID (for example, lower incidence compared with White ethnicity for Chinese or other ethnic groups apart from the post-COVID diagnosis cohort, and for Asian or Asian British only in the post-vaccination cohort). Similarly, there were inverse associations with coded long COVID of current smoking compared with never smoking, and positive associations with number of previous GP–patient interactions, across the four cohorts, although the magnitude of this association was lower in the post-COVID diagnosis and post-vaccination cohorts than in the primary and pre-vaccination cohorts. Associations with previous disease diagnoses were also broadly consistent across the four cohorts. Further, COVID-19 vaccination did not substantially modify associations of factors with coded long COVID-19, although it is likely to have substantially attenuated the overall incidence of COVID-19.^19^

### Strengths and limitations

A key strength of this study is its use of the data from the OpenSAFELY-TPP platform, which includes more than 40% of the English population.^20^ We analysed data from all eligible adults with follow-up of up to 26 months. The prevalence of coded long COVID was higher in people registered in an NHS primary care GP using EMIS EHR software than in practices using TPP software.^6^ The type of EHR software used is geographically clustered.^21^ However, we were not able to access data from practices using EMIS software.

The prevalence of coded long COVID in English primary care records was substantially lower than that found in population surveys. There is likely to be considerable under-ascertainment of long COVID in these records owing to difficulties in accessing care during the pandemic. Future research can investigate if access to free-text records might help decrease under-ascertainment.^22^ However, free text was not available for our analyses.

Fully aHRs quantify the contribution of each patient characteristic to predicting the outcome, having accounted for the value of each other patient characteristic. However, they do not have causal interpretations, because they do not distinguish between adjustment for confounders and mediators. Such misinterpretation of multiple adjusted effect estimates presented in a single table has been referred to as the 'table 2 fallacy'.^23^

As described in the 'COVID-19 diagnosis' section, we used data from multiple sources to capture COVID-19 diagnosis as accurately as possible. Patient characteristics were determined by using primary care records, and additional data from secondary care can improve the completeness and updated analysis can be conducted.

In the pre-vaccination cohort, follow-up was censored at the time of vaccination. Such censoring could lead to bias if it was informative, which would be the case if the incidence of coded long COVID differed systematically between people who were and were not vaccinated, having accounted for baseline covariates. We believe that our analyses adjusted for the major predictors of COVID-vaccination (for example, age, sex, ethnicity, IMD), which should have limited informative censoring, but cannot exclude the possibility that estimated associations were biased because of the censoring.

### Comparison with existing literature

Similar to other studies,^7,24,25^ we found positive associations of coded long COVID with female sex, obesity, mental health conditions, and living in less deprived areas. The latter association contrasts with the increased risk of SARS-CoV-2 infection with increasing deprivation, and illustrates the distinction between long COVID and coded long COVID, which depends on the ability of people with long COVID to access health care for their condition at a time of extreme pressure on health services. A previous EHR analysis also found that people living in less deprived areas had higher incidence of coded long COVID. However, in the same study, the analysis of longitudinal cohort studies found no association between IMD and self-reported long COVID.^7^

Among the general population, the incidence for coded long COVID was lower in people of Black ethnicity, similar to a previous study.^7^ We found similar incidence of coded long COVID in Asian and Asian British people and people of White ethnicity. Additionally, the incidence of coded long COVID was lower in Chinese or other ethnic groups, compared with people of White ethnicity. In general, the incidence of coded long COVID was higher in ever smokers but lower in current smokers, compared with never smokers. A previous study^7^ included only two categories for smoking status, and found no difference in the incidence of coded long COVID between current smokers and non-smokers. Smoking status in EHR may not be up to date, especially for people who had less frequent interaction with their GP.

A study in Moscow identified that pre-existing hypertension was associated with higher risk of long COVID 12 months since discharge from hospitalisation.^26^ Our fully adjusted model in the primary cohort showed that the incidence of coded long COVID was lower for people with a history of diagnosed hypertension, although the incidence was higher when only adjusted for age and sex. In other three cohorts, no association with hypertension was observed from the fully adjusted models. In the Moscow study, long COVID was assessed by clinicians after hospitalised COVID, while our study relied on people getting access to their GP and the diagnosis then being recorded.

A previous report to the UK Government’s Scientific Advisory Group for Emergencies found that the risk of coded long COVID was higher in adults with hospitalised than non-hospitalised COVID-19.^27^ Our study was restricted to adults. Other studies report that hospitalised COVID was also associated with higher risk of long COVID in children.^25,28,29^ A systematic review of 20 studies, identified higher risk of long COVID with female sex, mental health conditions, fatigue, and acute disease severity with respiratory symptoms.^30^

### Implications for practice

A potentially large proportion of people with long COVID did not have a long COVID code in their health records. The incidence of coded long COVID was influenced by the frequency of previous GP–patient interaction. Despite controlling for this factor, we identified a set of patient characteristics associated with coded long COVID, including sociodemographical variables, history of diagnosed diseases, and SARS-CoV-2 severity.

### Information governance

NHS England is the data controller for OpenSAFELY-TPP; TPP is the data processor; all study authors using OpenSAFELY have the approval of NHS England.^31^ This implementation of OpenSAFELY is hosted within the TPP environment which is accredited to the ISO 27001 information security standard and is NHS IG Toolkit compliant.^32^

Patient data has been pseudonymised for analysis and linkage using industry standard cryptographic hashing techniques; all pseudonymised datasets transmitted for linkage onto OpenSAFELY are encrypted; access to the platform is via a virtual private network (VPN) connection, restricted to a small group of researchers; the researchers hold contracts with NHS England and only access the platform to initiate database queries and statistical models; all database activity is logged; only aggregate statistical outputs leave the platform environment following best practice for anonymisation of results such as statistical disclosure control for low cell counts.^33^

The service adheres to the obligations of the UK General Data Protection Regulation (UK GDPR) and the Data Protection Act 2018. The service previously operated under notices initially issued in February 2020 by the Secretary of State under Regulation 3(4) of the Health Service (Control of Patient Information) Regulations 2002 (COPI Regulations), which required organisations to process confidential patient information for COVID-19 purposes; this set aside the requirement for patient consent.^34^ As of 1 July 2023, the Secretary of State has requested that NHS England continue to operate the Service under the COVID-19 Directions 2020.^35^ In some cases of data sharing, the common law duty of confidence is met using, for example, patient consent or support from the Health Research Authority Confidentiality Advisory Group.^36^

Taken together, these provide the legal bases to link patient datasets on the OpenSAFELY platform. GP practices, from which the primary care data are obtained, are required to share relevant health information to support the public health response to the pandemic and have been informed of the OpenSAFELY analytics platform.
